# Hurting all the way: The emotional antecedent and consequence of social rejection

**DOI:** 10.3389/fpsyg.2022.885384

**Published:** 2022-09-02

**Authors:** Xiaoying Wang, Miaomiao Li

**Affiliations:** ^1^School of Economics and Management, Tongji University, Shanghai, China; ^2^School of Economics and Management, Shanghai University of Political Science and Law, Shanghai, China

**Keywords:** social rejection, envy, social comparison, gender, negative affect

## Abstract

Social rejection is cold and hurtful, but how and why it is formed remains under-investigated. Our study offers one possible explanation from the rejector’s perspective by developing a moderated mediation model on the emotional antecedent and consequence of social rejection. Specifically, envious individuals use social rejection to complement their inferiority, further triggering their negative affect. Drawing on social comparison theory, we conducted an experience sampling methodology (ESM) investigation of 55 frontline workers through a 10-workday-survey (Level 1 *n* = 515). As predicted, daily envy is positively associated with daily social rejection. Daily social rejection is positively related to daily negative affect. Furthermore, daily social rejection mediates the relationship between daily envy and daily negative affect. These effects are more robust for females than males, including the impact of envy on social rejection and the impact of envy on negative affect via social rejection. We suggest the recipient and the rejector make psychological and behavioral adjustments accordingly. We also recommend that future research extend our current study methodologically and theoretically.

## Introduction

Individuals experience rejecting and being rejected in daily life with bitter feelings (Haldorai et al., 2022). Social rejection refers to the state where the rejector denies the request of the target in social interaction (Leary et al., 1998; Freedman et al., 2017). The antecedents of social rejection are diverse and complicated, as personalities (Killian et al., 2021; Rudert et al., 2021; Yaakobi, 2021; Redmond et al., 2022; Scott et al., 2022), perceptions (Zhang et al., 2022), status characteristics (Chung et al., 2021; Norman et al., 2021; Landini, 2022; Saco, 2022), and situational factors (Li Q. et al., 2021; Rudert et al., 2021; Graupmann and Pfundmair, 2022; Liborio et al., 2022) all play important roles in shaping social rejection. Generally speaking, social rejection happens to socially-unfavorable individuals or under difficult times. The ramifications of these situations tend to be hurtful to the target of rejection (i.e., the recipient or victim) (Haldorai et al., 2022). It is well-known that painful consequences such as aggression (Malamut et al., 2022; Rajchert et al., 2022), depression (Kwon and Jung, 2021; Wang et al., 2021), and decreased well-being (Chung et al., 2021; Jiang and Poon, 2021) are found among recipients, depicting the harmful nature of social rejection to the targets. Since human beings, as social animals, are inclined to avoid hurting others, why social rejection still prevails remains ambiguous (Legate et al., 2013).

Individuals conduct social rejection to win self-identification by their surroundings (Festinger, 1954; Buunk and Gibbons, 2007; Gerber, 2018; Gerber et al., 2018). The acceptance of socially-unfavorable individuals would indicate inferiority in their social status. Moreover, people have to isolate the target following other rejectors unwillingly for fear that they would otherwise be excluded by these rejectors (Legate et al., 2013). Furthermore, individuals feel under threat and therefore reject others. The threat could be occasional, such as the COVID-19 pandemic (Li Q. et al., 2021; Graupmann and Pfundmair, 2022), which has been studied in past researches; or it could also be status-related, i.e., arising from the superiority of the target, which is underexplored in the current cases.

Following this logic, we aim to offer an alternative explanation of the rejector’s motivation in social rejection from the perspective of status threat. We have focused on the rejector’s emotional clue of social rejection. As one of the symmetric parties in social rejection, the rejector feels the pain due to the loss of autonomy when s/he makes social rejection following his/her group (Legate et al., 2013). Also, an independent rejector is reported to have distressed feelings (Doolaard et al., 2020), guilt, and loss of relatedness after social rejection (Poulsen and Kashy, 2012). To further explicate the motivation and feeling when the rejector decides to perpetuate social rejection, we consider the social comparison theory, as it articulates well the psychological mechanism of status threat and relevant responses (Festinger, 1954; Buunk and Gibbons, 2007; Gerber, 2018; Gerber et al., 2018). Social comparison happens to every individual, especially those sensitive to others (Li M. M. et al., 2021). The competitiveness of contemporary society has further triggered social comparison between people (Wang et al., 2022), which may inevitably lead to envy. As the negative emotion triggered by others’ good fortune (Tai et al., 2012), envy may arouse one’s social rejection and further have emotional impacts on oneself.

More specifically, status threat-related social rejection may also vary with gender, a specific status characteristic; there is evidence that gender differences exist in the emotional responses of individuals to social rejection (Freedman et al., 2019; Rajchert et al., 2022). Hence, we set gender as a moderator to study gender differences in social rejection. Given that the emotional aspects of social rejection might be subtle and hard to identify, we adopt an experience sampling methodology (ESM) in this research, collecting the data on a daily basis in the consecutive ten-workday survey. This methodology also compensates for other lab studies regarding the rejector’s emotion (Poulsen and Kashy, 2012; Legate et al., 2013; Doolaard et al., 2020). We integrate theories from social comparison and gender characteristics to elucidate mechanisms linking envy, social rejection, negative affect, and gender using the ESM in our study. We aim to explore that social rejection is a response to envious individuals facing status threats during the upward social comparison process.

## Theory and hypotheses

### Overall framework

**FIGURE 1:**
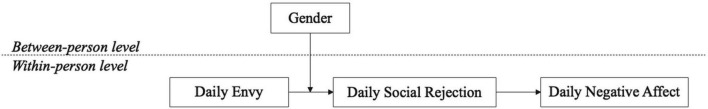
Theoretical framework for social rejection.

Social rejection is a form of social denial in building interactions among individuals (Leary et al., 1998; Freedman et al., 2017). Although undesirable, social rejection is still found in many circumstances for status-related purposes (Chung et al., 2021; Norman et al., 2021; Landini, 2022; Saco, 2022). Given that past research has already provided references to social rejections among inferior targets, we focus on social rejection toward superior recipients. Our basic assumption is that superior individuals may elicit status threats to the rejector based on social comparison and therefore be rejected. The status threat, derived from the upward social comparison and emotionally presented in the form of envy, may trigger social rejection (Lee and Duffy, 2019). The emotional consequence of upward social rejection is negative, similar to other categories of social rejection identified. Moreover, we regard gender, a specific status characteristic, as one boundary condition of upward-comparison-based social rejection. Accordingly, we propose a model of the emotional antecedent and consequence of social rejection. Figure 1 shows our proposed model.

### Envy and social rejection

Envy refers to the painful emotion aroused by others’ good fortune (Tai et al., 2012; Koopman et al., 2020). Individuals are likely to experience envy because of social comparison; when they feel inferior to others, they face a status threat that undermines their relative advantageous position. The inferiority could be related to different status characteristics such as demographics (Ahn et al., 2021; Javakhishvili et al., 2021), social status (Bollo et al., 2020), performance (Lee and Duffy, 2019; Sun et al., 2021), and competitiveness (Reh et al., 2018; Yu et al., 2018). This inferiority relates people to a worse self-image. Comparing oneself with superior others may threaten and impair one’s social status (Gaviria et al., 2021). People may benefit from such comparison by developing themselves to earn a better social status while getting hurt by others’ superiority (Lee and Duffy, 2019; Yang and Tang, 2021; Montal-Rosenberg and Moran, 2022).

Social rejection delineates one situation where an individual denies a request from the target in the social interaction (Leary et al., 1998; Freedman et al., 2017). According to Robinson et al. (2013), two related constructs of social rejection are social exclusion and ostracism. Social exclusion is the fact that one is excluded or devalued from desired relationships or by desired relationship partners or groups (MacDonald and Leary, 2005), while workplace ostracism depicts one’s subjective perception of being excluded at work (Ferris et al., 2008). In this paper, we use social rejection to describe the phenomenon that the rejector turning the recipient (or recipient’s request) down in the social interaction. Current literature has revealed that social rejection is an alternative for inferior individuals: their inferiority could be based on an inferior social status indicated by demographic factors and other status characteristics (Chung et al., 2021; Norman et al., 2021; Landini, 2022; Saco, 2022).

Social rejection’s negativity is mainly related to one of human’s basic needs: the need for relatedness (Ryan and Deci, 2000; Haldorai et al., 2022; Lin and Fan, 2022). A downward social rejection leads to destructive consequences for the recipient because it denies accessibility to a group and produces the feeling of loneliness. This denial could further trigger the recipient’s pro-social behavior (Haldorai et al., 2022) or aggression (Malamut et al., 2022; Rajchert et al., 2022). Social rejection may violate justice and moral stance, as rejection is seen as deviance from social norms (Poulsen and Kashy, 2012). For instance, individuals are forced to conduct social rejection under the pressure of conformity to satisfy their need to belong to their group, even though they may feel guilt for rejecting innocent others (Legate et al., 2013). In addition to group social rejection, personal social rejection could also lead to immoral feelings though necessary to conduct in some way (Freedman et al., 2019). To fill in the gap in the upward social rejection of the current research, we explore envy-induced social rejection, a typical occasion of upward social rejection, using social comparison theory.

Social comparison theory proposes that humans tend to compare with others to maintain a stable and accurate self-assessment, self-esteem, and self-worth, especially when objective information is unavailable or ambiguous (Greenberg and Pyszczynski, 1985; Taylor and Lobel, 1989; Aspinwall and Taylor, 1993; Suls et al., 2000; Gerber, 2018). People tend to conduct social comparisons with better-off individuals, leading to assimilation or contrast. Individuals conduct upward social comparison because they want a better self and are unsatisfied with their status quo. Their comparison target might have certain advantages perceived as inequity or unattainable for the comparer, such as being born with a silver spoon in the mouth or getting a straight-A at school. In this way, people may feel pain at others’ good fortune, as the good fortune is neither accessible nor legitimate for the comparer (Tai et al., 2012). The pain is particularly evident when the envier perceives that they should earn the same life as their target (Ferreira and Botelho, 2021). To further avoid such pain, individuals may reduce the connections with the superior target through social rejection: out of sight, out of mind.

Also, social rejection prevents self-depletion and promotes self-development (Li Q. et al., 2021). Fundamentally, competence is one of human’s basic psychological needs (Ryan and Deci, 2000). When interacting with the envied individuals, the envier might feel relative deprivation since their life expectations are realized by others, thwarting their needs for competence (Dineen et al., 2017). Avoiding contacts is one way to prevent further self-depletion from the need-threat perspective. It creates a safe psychological condition for self-development, particularly for highly self-critical individuals (Santor and Yazbek, 2006; Tai et al., 2012; Li M. M. et al., 2021).

Further, social rejection is a way to develop an independent identity for individuals, which might be conducive to improving one’s social status. The more the target’s advantages are perceived as unattainable, the more likely individuals may have to develop their specialties or skills to form their own identity. Social rejection categorizes oneself into a distinct category other than their comparing target. This contrast may help individuals relieve the pain caused by upward comparison. It may further form a unique self-construct and self-worth for the comparers in contrast to their competitors and do better than their competitors who are slightly better off in the following competitions. Thus, we propose the hypothesis:

*Hypothesis 1: Daily envy is positively associated with daily social rejection.*

### Social rejection and negative affect

Social rejection keeps the envier at a distance from their competitors to avoid status threats. However, this threat-avoidance behavior might generate negative affect equally for the recipient and rejector (Doolaard et al., 2020). Past research has observed negative affect among recipients (Stinson et al., 2011; Hebl et al., 2012; Li et al., 2012; Kawamoto et al., 2017; Miyagawa et al., 2021). As the symmetric party in social rejection, the emotional responses of the rejector still lack awareness from scholars.

Previous research reveals that social rejection could lead to the rejector’s negative feelings. First of all, social rejection is a behavior that violates basic social norms (Poulsen and Kashy, 2012). The rejector feels guilt for not accepting others’ requests (Ciarocco et al., 2001; Bastian et al., 2013; Legate et al., 2013). This rejection also deprives people’s relatedness, generating negative feelings as their need to belong is unsatisfied (Ryan and Deci, 2000). The rejector’s psychological resource is depleted during this process, although the behavior is intended to save energy for self-development (Baumeister et al., 1998; Inzlicht and Schmeichel, 2012; Mawritz et al., 2017).

The rejection takes away the opportunity to access and assimilate with superior individuals through interaction. Therefore, the rejector is not identified as a superior member of the recipient’s group. Meanwhile, they lose human and social capital from the recipient (Lee and Duffy, 2019). The loss, which the rejector could have avoided, might be recognized and cause the rejector’s psychological discomfort due to their fear of resource loss (Hobfoll, 1989; Hobfoll et al., 2018). As human capital and social capital are critical to one’s self-development, the rejector may find it unworthy to sacrifice the human capital and social capital at the cost of self-recovery by themselves. Taking all aspects into consideration, we propose the following hypothesis:

*Hypothesis 2: Daily social rejection is positively associated with daily negative affect.*

### Mediating effect of social rejection

As an undesirable form of social interaction, social rejection is universal in daily lives. In the upward social comparison, social rejection is a status threat-related response elicited by envy (Breidenthal et al., 2020). As a consequence of seeing other’s superiority, individuals have negative feelings toward themselves: They feel stressed about getting behind and unable to achieve what others already have, and depressed about the perceived unfairness in the way good fortune is distributed (Dineen et al., 2017; Pan et al., 2021; Tussing et al., 2022). The rejector is not intended to take social rejection as deviant behavior but as a way of hiding or releasing the psychological burden caused by status threats in the social interaction. Social rejection is performed as one process of emotional manifestation; it is both the agent and the approach. After social rejection, the rejector’s negative affect increases due to self-loss and hurting the target of their rejection. Thus, we propose the following hypothesis:

*Hypothesis 3: Daily social rejection mediates the relationship between daily envy and daily negative affect.*

### Moderating effects of gender

Social rejection based on the upward comparison may be closely related to status characteristics, one way to reflect people’s social status. A typical status characteristic affecting the level of social rejection, as depicted in the previous article, is gender (Freedman et al., 2019). Although meta-analysis does not demonstrate any gender differences in envy (Li M. M. et al., 2021), it is shown that women face a more comprehensive range of social comparisons, including appearance (Lewis and Simpson, 2012) and body image (Kiefer et al., 2006). As women embrace a higher level of communal characteristics (Schock et al., 2019), they are more environment-dependent when making self-identifications. Therefore, they are more likely to compare themselves to others, recognize others’ goodness in various social comparisons and feel envious than men. The envious state may drive women to reject those better-off others as the temporal maintenance of a stable psychological state. Under such circumstances, women tend to conduct social rejection more than their male counterparts. Consequently, we propose the following hypothesis:

*Hypothesis 4: Gender moderates the relationship between daily envy and daily social rejection. Compared with male, female reports a stronger relationship between daily envy and daily social rejection.*

Envious female is prone to conduct more social rejection, further experiencing more negative affect in the social comparison process of envy (Festinger, 1954). Social rejection has the potential benefits of maintaining one’s psychological stability in the short run. However, the ultimate emotional consequence of social rejection tends to be negative for females compared with males since envy and social rejection deviate from women’s social gender norms of warmth and consideration (Vial et al., 2018; Freedman et al., 2019). In alignment with the immoral stand taken by envy and social rejection, females may be more likely to fall into negative affect than males (Freedman et al., 2019). The behavior of social rejection may indicate an inferior coping of upward social comparison and status threat and, finally, turn into a women’s self-blame for its social deviance. The blame could provoke more negative affect on females than male counterparts (Brescoll, 2016; Gupta et al., 2018; Schock et al., 2019). Moreover, women tend to display more altruism and philanthropical behaviors. The action of social rejection may violate their gender characteristics and lead to negative affect such as women’s guilt for not being considerate as usual. Some women may also consider social rejection from a moral perspective and regret this “immoral” behavior (Freedman et al., 2019). Therefore, we propose the following hypothesis:

*Hypothesis 5: Gender moderates the mediating mechanism of social rejection on the relationship between daily envy and daily negative affect. Compared with male, female reports a stronger effect on the mediating mechanism of social rejection on the relationship between daily envy and daily negative affect.*

## Materials and methods

### Sample and procedures

To study the emotional antecedent and consequence of social rejection, we conduct a 3-week daily data collection in the electronics factory on the eastern coast using an experience sampling methodology (ESM), following the procedure of Fisher and To (2012). With the support of senior leaders, every participant voluntarily reports their daily emotions and behaviors. All participants were asked to complete an electronic survey within the notifications by phone on a daily basis. We collected data every day between 3:00 and 5:00 pm (working hours) in the 10-workday survey. All the 70 participants are frontline workers of the same working status. They work with their coworkers almost daily, have frequent contact with them, and are close to them. Sixty percent of our participants are male, and 40% are female. Fifty-five participants have completed the survey for at least three full days, remaining 78.57% valid data. These participants are 22 years old on average. Most of them (above 85%) are newcomers and have a Bachelor’s degree.

Every individual is required to report their emotions and behaviors every day. Specifically, they report their perception of envy, social rejection, and negative affect. Envy on day 1 predicts social rejection and negative affect on day 1. We added the power analysis to justify the sample size by *R* procedure, which is acceptable and enough. We applied Chi-square tests to detect an effect of a given size with a given degree of confidence to report the required sample size. For the power of the Chi-square tests, when the total sample size is 515, the degree of freedom is 54. The effect size is moderate (0.3), and a significance level of 0.01 is employed, calculating the sample size by *R* to obtain a power of 0.874, which is higher than 0.80 and indicates enough power.

### Measures

We follow the procedure of translation and back-translation. All responses were on a 5-point Likert-type scale from 1 (strongly disagree) to 5 (strongly agree).

#### Daily envy

Participants rated envy using a 5-item scale adapted by Vecchio (2005). The items were “Today, I feel most of my coworkers have it better than I do”; “Today, I feel my supervisor values the efforts of others more than he/she values my efforts”; “Today, I feel that I’ll never have a job as good as some that I’ve seen”; “Today, I don’t know why, but I seem to be the underdog at work”; and “Today, it is somewhat annoying to see others have all the luck in getting the best assignments.” The average alpha coefficient for these five items was 0.926.

#### Daily social rejection

Participants rated social rejection with a 10-item scale adapted by Ferris et al. (2008). Sample items were “Today, I ignored envied target at work”; “Today, I left the area when the envied target entered”; and “Today, the envied target’ greetings have gone unanswered at work from me.” The average alpha coefficient for these ten items was 0.957.

#### Daily negative affect

Participants rated their negative affect using the 10-item scale developed by Watson et al. (1988). A sample item was “Today, I feel upset.” The average alpha coefficient for these ten items was 0.957.

Gender, as a level-2 construct, was coded as 1 for male and 2 for female. We test the moderating effect of gender on the relationship between social rejection and emotions.

## Analytic approach

We apply Mplus 7.4 to test the multilevel path analysis of the hypothesized model in Figure 1, considering the multilevel structure of the data (days and persons). First, we verified that there was sufficient within-individual variability to justify multilevel analysis (Podsakoff et al., 2019). There was substantial within-person variance: daily envy, 41.27%; social rejection, 35.33%; negative affect, 34.21%. Second, we centered the predictors of daily envy by group-mean and calculated the product of daily envy and gender. Third, we used a bootstrap procedure with 20,000 iterations to assess the mediation effect and estimate the bias-corrected confidence intervals (CIs) based on the Monte Carlo method (Preacher and Selig, 2012). Further, we checked the significance of the difference in this indirect effect at higher and lower levels of gender (±SD) (Hayes, 2015). In particular, we provided data and code on OSF in the following linkage^1^, including all data analysis steps and figures, to advance open science practices.

At Level-1 of the two-level model, we specified random effects of daily envy, daily social rejection, and daily negative affect. At Level-2, we specified the cross-level moderating effect of gender on the random slope between daily envy and daily social rejection and the cross-level main effect of gender on daily social rejection. Daily envy, daily social rejection, and daily negative affect were all group-mean centered on obtaining unbiased estimates (Hofmann and Gavin, 1998; Liu et al., 2015).

## Results

Before testing the hypotheses, we ran a multilevel confirmatory factor analysis of the four focal variables in Figure 1 (gender, daily envy, daily social rejection, and daily negative affect). This model exhibited good fit, χ^2^(93) = 154.97 (*p* < 0.01); CFI = 0.985; TLI = 0.980; RMSEA = 0.036; SRMR within = 0.026; SRMR between = 0.060, supporting the construct distinctiveness of our variables. As shown in Table 1, we report the means, standard deviations, and correlations of the variables, supporting the hypothesized model.

**TABLE 1:** Means, standard deviations, and correlations of the study variables for the hypothesized model.

	Mean	*SD*	1	2	3	4
**Level-1 variables**						
(1) Daily envy	2.28	0.83				
(2) Daily social rejection	2.38	0.88	0.227**			
(3) Daily negative affect	2.13	0.87	0.326**	0.284**		
**Level-2 variables**						
(4) Gender	1.37	0.48	−0.073	−0.169**	−0.050	

Level-1 *n* = 515; level-2 *n* = 55. Level-1 exogenous variables were centered at each person’s mean.

*p < 0.05, **p < 0.01.

Hypothesis 1 proposed that daily envy is positively related to social rejection, which is supported by the results in Table 2 (γ = 0.51, *p* < 0.01). Further, daily social rejection is positively associated with daily negative affect (γ = 0.46, *p* < 0.01), supporting Hypotheses 2. Hypotheses 3 examined the mediating effect of daily social rejection. The results in Table 3 show that daily envy was positively associated with daily negative affect via daily social rejection (estimate = 0.059, 95% CI [0.0130, 0.0898]). Thus, Hypotheses 3 were supported.

**TABLE 2:** Multilevel path analysis results for the hypothesized model.

	Daily social rejection				Daily negative affect					
Predictor	γ	*SE*	γ	*SE*	γ	*SE*	γ	*SE*	γ	*SE*
Intercept	2.38**	0.03	2.71	0.10	0.51**	0.08	1.03**	0.10	0.28**	0.09
**Level-1 predictors**										
Daily envy	0.51**	0.04	−0.06	0.12	0.71**	0.03			0.62**	0.04
Daily social rejection							0.46**	0.04	0.18**	0.04
**Level-2 predictors**										
Gender			−0.23**	0.07						
**Cross-level moderator**										
Daily envy * Gender			0.42**	0.09						

Level-1 *n* = 515; level-2 *n* = 55. Level-1 exogenous variables were centered at each person’s mean. SE, standard error.

*p < 0.05, **p < 0.01.

**TABLE 3:** Results of indirect and conditional indirect effects from the multilevel path analysis.

Indirect effect	Gender	Estimate	95% CI
Daily envy → Social rejection → Negative affect		0.059	[0.0130, 0.0898]
	Female	0.089	[0.0281, 0.1523]
	Male	0.026	[0.0275, 0.1524]
	Difference	0.063	[0.0095, 0.107]

Level-1 *n* = 515; level-2 *n* = 55. The CIs of the bias-corrected indirect effects and conditional indirect effects are based on 20,000 Monte Carlo bootstrap samples. All of the indirect effects were calculated, accounting for direct effects. Unstandardized effects are reported in the table. CI, confidence interval.

**FIGURE 2:**
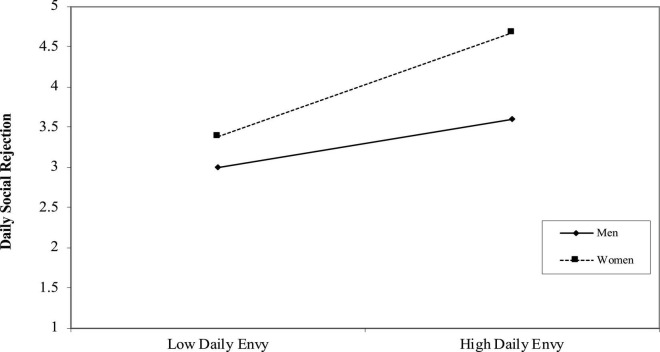
The moderating effect of gender.

We examined whether gender, as a between-level variable, would moderate the within-individual, direct effect of daily envy and daily social rejection and the indirect impact of daily envy on daily negative affect through daily social rejection. Tables 2, 3 show the results of our analyses. They reveal that gender had a cross-level buffering moderating effect on the relationship between daily envy and daily social rejection (*b* = 0.42, *p* < 0.01). Further, Figure 2 shows the significance of the moderating effect, supporting Hypotheses 3.

The indirect effect of daily envy on daily negative affect via daily social rejection was significant at higher levels of gender (estimate: 0.089; 95% CI [0.0281, 0.1523]) and at lower levels (estimate: 0.026; 95% CI [0.0275, 0.1524]), which indicated significant difference in the indirect effect (estimate: 0.063; 95% CI [0.0095, 0.107]). These results supported Hypotheses 4.

## Discussion

We examined the emotional antecedents and consequences of social rejection on a daily basis by using the experience sampling method. Focusing on the rejectors’ perspective, we found that daily envy triggers individuals to conduct social rejection, then induces more negative affect. Compared with envious males, envious females report a higher level of social rejection and, in turn, generate more negative affect. Our results support the moderated mediation model.

### Theoretical implications

The study has four main theoretical implications. First, we deploy the ESM to capture how individuals’ envy influences their level of social rejection and the consequent negative affect on a daily basis following the methodology procedure of Fisher and To (2012), which was widely used in the current studies on emotions and behaviors (Koopmann et al., 2019; Tang et al., 2022). Our study compensates for the current research field as few empirical studies have investigated the nature of social rejection through a dynamic methodology. Experience sample modeling has enabled us to record fluctuations in social rejection by day-to-day monitoring, which generates more credible results than lab testing or the traditional longitudinal study methodology.

Second, the study extends the literature on social rejection from the rejector’s perspective by answering why individuals conduct social rejection and their subsequent feelings. Specifically, we test the relationship between envy, social rejection, and negative affect, articulating the psychological mechanism of social rejection from the rejector’s emotional perspective. As a negative response to others’ good fortune from an ambitious individual, envy is positively related to social rejection, and negative affect follows. Our results are consistent with Poulsen and Kashy (2012); Legate et al. (2013), and Doolaard et al. (2020), which all acknowledge that proactive and reactive social rejection would lead to the rejector’s negative affect. We further explore the emotional clue triggering social rejection and depict how such negative emotion (envy) is passed through social rejection and elicits further negative affect. People facing status threats may take social rejection as one self-protection mechanism. However, such measure does not eliminate and sometimes even generate additional negative affect by immoral feelings, loss of capital, and unsatisfied need to belong.

Third, while most studies on social comparison theory investigate downward social comparison (Haldorai et al., 2022; Malamut et al., 2022; Rajchert et al., 2022), this study compensates well by studying upward social comparison. We propose that social rejection is one way people prevent themselves from self-depletion while seeing others’ goodness in the upward social comparison. Past research on upward social comparison is conducted in the social interaction context, where the comparer responds to such comparisons by pro-social and anti-social behaviors (Pan et al., 2021; Yang and Tang, 2021; Boecker et al., 2022). Our study investigates the situation where individuals take an avoidance attitude toward upward social comparison by denying participation via social rejection. Our results show that avoiding social interaction does not prevent individuals from negative feelings in upward social comparison. The core factor that forms those feelings is the threat evoked by the rejector’s social status and self-worth during upward social comparison, which causes the feeling of lacking competence and a subsequent feeling of lacking relatedness caused by social rejection.

Fourth, our study contributes to gender research. As one prominent status characteristic, female appears to be a distinct status characteristic in the current research (Ecker et al., 2020; Farh et al., 2020). Scholars have shown that women tend to denigrate one another due to limited opportunities for upward mobility in their organizations (Parks-Stamm et al., 2008; Derks et al., 2011; Derks et al., 2016; Haas et al., 2016; Arvate et al., 2018). Also, women are reported to contrast to envied targets by distancing themselves from similar others rather than male peers (Elmagrhi et al., 2019; Kurt Yilmaz and Surgevil Dalkilic, 2019). We extend the current gender research field by discovering that women tend to be more self-isolated from social interactions than their male counterparts. Their rejection is not only toward same-gender peers for a better self-identity but in a broader sense to all individuals who are better off than them. This behavior does not improve women’s gender status or self-worth but violates their gender role and self-image as social rejection serves as deviance to gender norms.

### Practical implications

The study shows that social rejection hurts the rejector. It might not be economical for the rejector to conduct social rejection since such rejection would neither improve an individual’s social status and self-worth nor change their negative emotions. By contrast, the rejector may have self-adjustment if they meet competitive peers in the social environment. They may change their view toward the target of upward social comparison and further take social interaction as an opportunity to expand their social network. Also, the rejector might recognize themselves as outstanding individuals to be better engaged in a competitive environment. The request for social interaction from the target is a sign of recognition for their competence and achievement; it is conducive and offers the opportunity for both the rejector and the recipient to learn and grow. By accepting the request, both parties could develop themselves and embrace a better self-image and social status. Lastly, the rejector may also stop being ashamed of taking social rejection as it is one way to release their pain and maintain physiological and mental health.

For the recipient of social rejection, the study has indicated their social competitiveness as they are the winner of social comparison in the rejector’s mind. Considering this point, the recipient might feel more reasonable when rejected. Furthermore, the recipient might also pay attention to how the request is sent and their past social interactions with the rejector to improve their social skills. In addition, the recipient could take advantage of their competitive advantage to help individuals in need around them proactively. In this case, their advantage may be beneficial for expanding their social networks and reducing other people’s hostility in advance. Besides, the recipient could also be more authentic than simply conducting impression management. A perfect person is unreal, and a perfect personality is interpreted as very aggressive in social competition. The recipient might ask for other people’s help when getting in trouble instead of figuring it out alone. This action may provide more happiness to the recipient to save energy and feel the warmth from others.

### Limitations and directions for future research

Despite our efforts in designing methods and conducting analyses, this study has three limitations. First, we collected the data from the same source, which may raise concerns about common method variance (Podsakoff et al., 2012). Future research can take other-reported measures for social rejection. For instance, the rejectors’ rate of their social rejection in the workplace could be rated by their coworkers instead. Second, a potential concern exists regarding control variables. We only measured negative affect and ignored positive affect. In effect, positive affect could be considered as a control variable. Future research may also consider other control variables, which could help develop a solid study about social rejection and emotions. Third, we used negative affect as one construct to detect the rejector’s feelings after conducting social rejection out of envy. To have a more profound understanding of social rejection’s emotional consequences, we recommend that future research explore specific emotions like sadness, anger, or frustration to identify how social rejection is linked to each emotional reaction. In addition, future research could also explore self-protective-related antecedents to explore beyond aggressive factors such as envy. Fourth, we use survey data to make causal inferences, which might not be sufficient for solid evidence (Law et al., 2016), including the main effect and mediating effect of social rejection. Future research might conduct experimental studies as further evidence for the causal relationships. For example, scholars may conduct two time-lagged experimental studies to examine the main and mediating effects by strengthening the causal inferences. Finally, even though we have conducted the power analysis, we call for a more exact multilevel power analysis, such as powerlmm (Magnusson, 2018). As Gabriel et al. (2019) concluded, only 2 of the 107 ESM studies conducted a power analysis, showing that “power issues are rarely discussed in ESM research” (Gabriel et al., 2019, p. 975). Future research applying ESM should conduct power analysis.

### Conclusion

To test the emotional antecedents and consequences of social rejection, we use experience sample modeling to explore how daily envy drives individuals to use social rejection to complement their inferiority and finally triggers negative affect. Drawing on social comparison theory, we conducted an experience sampling methodology (ESM) investigation of 55 frontline workers through a 10-workday survey (Level 1 *n* = 515). As predicted, daily envy is positively associated with daily social rejection. Daily social rejection is positively related to daily negative affect. Furthermore, daily social rejection mediates the relationship between daily envy and daily negative affect. These effects are more prominent among females than males, including the impact of envy on social rejection and the impact of envy on negative affect via social rejection. We suggest the rejector stop being ashamed of taking social rejection and try to connect with others instead of rejecting the recipient to improve their self-image and social status. We also suggest that the recipient proactively improve communication skills and help people in need. The study extends the literature on social rejection, social comparison, and gender.

## Data availability statement

The raw data supporting the conclusions of this article will be made available by the authors, without undue reservation, to any qualified researcher.

## Ethics statement

The studies involving human participants were reviewed and approved by Tongji University. The patients/participants provided their written informed consent to participate in this study.

## Author contributions

XW and ML were responsible for idea generation and revised the manuscript. ML conducted material preparation, data collection, and analysis. XW wrote the first draft. Both authors commented on previous versions of the manuscript and read and approved the final manuscript.

## Footnotes

1
https://osf.io/trmsa/

## References

[B1] Ahn S. Ha Y. W. Jo M. S. Kim J. Sarigollu E. (2021). A cross-cultural study on envy premium: the role of mixed emotions of benign and malicious envies. *Curr. Psychol.* [Epub ahead of print]. 10.1007/s12144-021-01679-7

[B2] Arvate P. R. Galilea G. W. Todescat I. (2018). The queen bee: a myth? The effect of top-level female leadership on subordinate females. *Leadersh. Quart.* 29 533–548. 10.1016/j.leaqua.2018.03.002

[B3] Aspinwall L. G. Taylor S. E. (1993). Effects of social-comparison direction, threat, and self-esteem on affect, self-evaluation, and expected success. *J. Pers. Soc. Psychol.* 64 708–722. 10.1037/0022-3514.64.5.708 8505703

[B4] Bastian B. Jetten J. Chen H. Radke H. R. M. Harding J. F. Fasoli F. (2013). Losing our humanity: the self-dehumanizing consequences of social ostracism. *Pers. Soc. Psychol. B* 39 156–169. 10.1177/0146167212471205 23386654

[B5] Baumeister R. F. Bratslavsky E. Muraven M. Tice D. M. (1998). Ego depletion: is the active self a limited resource? *J. Pers. Soc. Psychol.* 74 1252–1265. 10.1037/0022-3514.74.5.1252 9599441

[B6] Boecker L. Loschelder D. D. Topolinski S. (2022). How individuals react emotionally to others’ (mis)fortunes: a social comparison framework. *J. Pers. Soc. Psychol.* [Epub ahead of print]. 10.1037/pspa0000299 35025600

[B7] Bollo H. Hager D. R. Galvan M. Orosz G. (2020). The role of subjective and objective social status in the generation of envy. *Front. Psychol.* 11:513495. 10.3389/fpsyg.2020.513495 33384633 PMC7770237

[B8] Breidenthal A. P. Liu D. Bai Y. T. Mao Y. N. (2020). The dark side of creativity: coworker envy and ostracism as a response to employee creativity. *Organ. Behav. Hum.* 161 242–254. 10.1016/j.obhdp.2020.08.001

[B9] Brescoll V. L. (2016). Leading with their hearts? How gender stereotypes of emotion lead to biased evaluations of female leaders. *Leadersh. Q.* 27 415–428. 10.1016/j.leaqua.2016.02.005

[B10] Buunk A. P. Gibbons F. X. (2007). Social comparison: the end of a theory and the emergence of a field. *Organ. Behav. Hum.* 102 3–21. 10.1016/j.obhdp.2006.09.007

[B11] Chung S. Kim M. Auh E. Y. Park N. S. (2021). WHO’s global age-friendly cities guide: its implications of a discussion on social exclusion among older adults. *Int. J. Environ. Res. Public Health* 18:8027. 10.3390/ijerph18158027 34360319 PMC8345595

[B12] Ciarocco N. J. Sommer K. L. Baumeister R. F. (2001). Ostracism and ego depletion: the strains of silence. *Pers. Soc. Psychol. B* 27 1156–1163. 10.1177/0146167201279008

[B13] Derks B. Ellemers N. van Laar C. de Groot K. (2011). Do sexist organizational cultures create the Queen Bee? *Br. J. Soc. Psychol.* 50 519–535. 10.1348/014466610x525280 21884548

[B14] Derks B. Van Laar C. Ellemers N. (2016). The queen bee phenomenon: why women leaders distance themselves from junior women. *Leadersh. Q.* 27 456–469. 10.1016/j.leaqua.2015.12.007

[B15] Dineen B. R. Duffy M. K. Henle C. A. Lee K. (2017). Green by comparison: deviant and normative transmutations of job search envy in a temporal context. *Acad. Manage. J.* 60 295–320. 10.5465/amj.2014.0767

[B16] Doolaard F. T. Lelieveld G. J. Noordewier M. K. van Beest I. van Dijk E. (2020). Get out or stay out: how the social exclusion process affects actors, but not targets. *J. Exp. Soc. Psychol.* 88:103946. 10.1016/j.jesp.2019.103946

[B17] Ecker A. Ennser-Jedenastik L. Haselmayer M. (2020). Gender bias in asylum adjudications: evidence for leniency toward token women. *Sex Roles* 82 117–126. 10.1007/s11199-019-01030-2

[B18] Elmagrhi M. H. Ntim C. G. Elamer A. A. Zhang Q. J. (2019). A study of environmental policies and regulations, governance structures, and environmental performance: the role of female directors. *Bus. Strateg. Environ.* 28 206–220. 10.1002/bse.2250

[B19] Farh C. I. C. Oh J. K. Hollenbeck J. R. Yu A. Lee S. M. King D. D. (2020). Token female voice enactment in traditionally male-dominated teams: facilitating conditions and consequences for performance. *Acad. Manage. J.* 63 832–856. 10.5465/amj.2017.0778

[B20] Ferreira K. Botelho D. (2021). (Un)deservingness distinctions impact envy subtypes: implications for brand attitude and choice. *J. Bus. Res.* 125 89–102. 10.1016/j.jbusres.2020.12.008

[B21] Ferris D. L. Brown D. J. Berry J. W. Lian H. (2008). The development and validation of the workplace ostracism scale. *J. Appl. Psychol.* 93 1348–1366. 10.1037/a0012743 19025252

[B22] Festinger L. (1954). A theory of social comparison processes. *Hum. Relat.* 7 117–140.

[B23] Fisher C. D. To M. L. (2012). Using experience sampling methodology in organizational behavior. *J. Organ. Behav.* 33 865–877. 10.1002/job.1803

[B24] Freedman G. Burgoon E. M. Ferrell J. D. Pennebaker J. W. Beer J. S. (2017). When saying sorry may not help: the impact of apologies on social rejections. *Front. Psychol.* 8:1375. 10.3389/fpsyg.2017.01375 28848484 PMC5554531

[B25] Freedman G. Fetterolf J. C. Beer J. S. (2019). Engaging in social rejection may be riskier for women. *J. Soc. Psychol.* 159 575–591. 10.1080/00224545.2018.1532388 30513066

[B26] Gabriel A. S. Podsakoff N. P. Beal D. J. Scott B. A. Sonnentag S. Trougakos J. P. (2019). Experience sampling methods: a discussion of critical trends and considerations for scholarly advancement. *Organ. Res. Methods* 22 969–1006. 10.1177/1094428118802626

[B27] Gaviria E. Quintanilla L. Navas M. J. (2021). Influence of group identification on malicious and benign envy: a cross-sectional developmental study. *Front. Psychol.* 12:663735. 10.3389/fpsyg.2021.663735 34276488 PMC8277992

[B28] Gerber J. P. (2018). “Social comparison theory,” in *Encyclopedia of Personality and Individual Differences*, eds Zeigler-Hill V. Shackelford T. K. (Cham: Springer International Publishing), 1–8.

[B29] Gerber J. P. Wheeler L. Suls J. (2018). A social comparison theory meta-analysis 60+years on. *Psychol. Bull.* 144 177–197. 10.1037/bul0000127 29144145

[B30] Graupmann V. Pfundmair M. (2022). When ostracism is mandated: COVID-19, social distancing, and psychological needs. *J. Soc. Psychol.* [Epub ahead of print]. 10.1080/00224545.2022.2026284 35102815

[B31] Greenberg J. Pyszczynski T. (1985). Compensatory self-inflation - a response to the threat to self-regard of public failure. *J. Pers. Soc. Psychol.* 49 273–280. 10.1037/0022-3514.49.1.273

[B32] Gupta V. K. Han S. Mortal S. C. Silveri S. Turban D. B. (2018). Do women CEOs face greater threat of shareholder activism compared to male CEOs? A role congruity perspective. *J. Appl. Psychol.* 103 228–236. 10.1037/apl0000269 29016162

[B33] Haas M. Koeszegi S. T. Zedlacher E. (2016). Breaking patterns? How female scientists negotiate their token role in their life stories. *Gender Work Organ.* 23 397–413. 10.1111/gwao.12124

[B34] Haldorai K. Kim W. G. Li J. (2022). I’m broken inside but smiling outside: when does workplace ostracism promote pro-social behavior? *Int. J. Hosp. Manag.* 101:103088. 10.1016/j.ijhm.2021.103088

[B35] Hayes A. F. (2015). An index and test of linear moderated mediation. *Multiv. Behav. Res.* 50 1–22. 10.1080/00273171.2014.962683 26609740

[B36] Hebl M. R. Williams M. J. Sundermann J. M. Kell H. J. Davies P. G. (2012). Selectively friending: racial stereotypicality and social rejection. *J. Exp. Soc. Psychol.* 48 1329–1335. 10.1016/j.jesp.2012.05.019

[B37] Hobfoll S. E. (1989). Conservation of resources - a new attempt at conceptualizing stress. *Am. Psychol.* 44 513–524. 10.1037//0003-066x.44.3.513 2648906

[B38] Hobfoll S. E. Halbesleben J. Neveu J. P. Westman M. (2018). Conservation of resources in the organizational context: the reality of resources and their consequences. *Annu. Rev. Organ. Psychol.* 5 103–128. 10.1146/annurev-orgpsych-032117-104640

[B39] Hofmann D. A. Gavin M. B. (1998). Centering decisions in hierarchical linear models: implications for research in organizations. *J. Manage.* 24 623–641. 10.1177/014920639802400504

[B40] Inzlicht M. Schmeichel B. J. (2012). What is ego depletion? Toward a mechanistic revision of the resource model of self-control. *Perspect. Psychol. Sci.* 7 450–463. 10.1177/1745691612454134 26168503

[B41] Javakhishvili N. Butsashvili N. Vardanashvili I. Gogibedashvili A. (2021). Social-structural antecedents come forward to elicit envy to distant out-groups. *Front. Psychol.* 12:610571. 10.3389/fpsyg.2021.610571 34135798 PMC8200634

[B42] Jiang Y. F. Poon K. T. (2021). Stuck in companionless days, end up in sleepless nights: relationships between ostracism, rumination, insomnia, and subjective well-being. *Curr. Psychol.* [Epub ahead of print]. 10.1007/s12144-021-01474-4

[B43] Kawamoto T. Ura M. Hiraki K. (2017). Curious people are less affected by social rejection. *Pers. Indiv. Differ.* 105 264–267. 10.1016/j.paid.2016.10.006

[B44] Kiefer A. Sekaquaptewa D. Barczyk A. (2006). When appearance concerns make women look bad: solo status and body image concerns diminish women’s academic performance. *J. Exp. Soc. Psychol.* 42 78–86. 10.1016/j.jesp.2004.12.004

[B45] Killian H. J. Lim S. L. Bruce J. M. Ha O. R. (2021). Social rejection influences prosocial sharing decision-making in inequality contexts. *Curr. Psychol.* [Epub ahead of print]. 10.1007/s12144-021-01963-6

[B46] Koopman J. Lin S. H. Lennard A. C. Matta F. K. Johnson R. E. (2020). My coworkers are treated more fairly than me! A self-regulatory perspective on justice social comparisons. *Acad. Manage. J.* 63 857–880. 10.5465/amj.2016.0586

[B47] Koopmann J. Johnson R. E. Wang M. Lanaj K. Wang G. F. Shi J. Q. (2019). A self-regulation perspective on how and when regulatory focus differentially relates to citizenship behaviors. *J. Appl. Psychol.* 104 629–641. 10.1037/apl0000366 30550300

[B48] Kurt Yilmaz B. Surgevil Dalkilic O. (2019). Conceptual framework about tokenism phenomenon in organizations. *Int. J. Contemp. Econ. Acad.* 9 205–231. 10.5281/zenodo.3537908

[B49] Kwon E. J. Jung H. S. (2021). The effect of labor and relationship exclusions on older korean men with depression. *Int. J. Environ. Res. Public Health* 18:5876. 10.3390/ijerph18115876 34070783 PMC8199037

[B50] Landini I. (2022). The exclusion of migrants and refugees from welfare programs in Austria: the “legitimizing explanations” across different policy areas. *Int. J. Sociol. Soc. Pol.* 42 159–176. 10.1108/Ijssp-10-2020-0486

[B51] Law K. S. Wong C. S. Yan M. Huang G. H. (2016). Asian researchers should be more critical: the example of testing mediators using time-lagged data. *Asia Pac. J. Manag.* 33 319–341. 10.1007/s10490-015-9453-9

[B52] Leary M. R. Springer C. Negel L. Ansell E. Evans K. (1998). The causes, phenomenology, and consequences of hurt feelings. *J. Pers. Soc. Psychol.* 74 1225–1237. 10.1037/0022-3514.74.5.1225

[B53] Lee K. Duffy M. K. (2019). A functional model of workplace envy and job performance: when do employees capitalize on envy by learning from envied targets? *Acad. Manage. J.* 62 1085–1110. 10.5465/amj.2016.1202

[B54] Legate N. DeHaan C. R. Weinstein N. Ryan R. M. (2013). Hurting you hurts me too: the psychological costs of complying with ostracism. *Psychol. Sci.* 24 583–588. 10.1177/0956797612457951 23447557

[B55] Lewis P. Simpson R. (2012). Kanter revisited: gender, power and (in)visibility. *Int. J. Manag. Rev.* 14 141–158. 10.1111/j.1468-2370.2011.00327.x

[B56] Li H. J. Zeigler-Hill V. Yang J. Jia L. Xiao X. Luo J. L. (2012). Low self-esteem and the neural basis of attentional bias for social rejection cues: evidence from the N2pc ERP component. *Pers. Indiv. Differ.* 53 947–951. 10.1016/j.paid.2012.03.004

[B57] Li M. M. Xu X. F. Kwan H. K. (2021). The antecedents and consequences of workplace envy: a meta-analytic review. *Asia Pac. J. Manag.* [Epub ahead of print]. 10.1007/s10490-021-09772-y PMC8365139 34408692

[B58] Li Q. Luo R. L. Zhang X. Y. Meng G. T. Dai B. B. Liu X. (2021). Intolerance of COVID-19-Related Uncertainty and negative emotions among chinese adolescents: a moderated mediation model of risk perception, social exclusion and perceived efficacy. *Int. J. Environ. Res. Public Health* 18:2864. 10.3390/ijerph18062864 33799731 PMC8002157

[B59] Liborio M. P. Martinuci O. D. Machado A. M. C. Lyrio R. D. Bernardes P. (2022). Time-space analysis of multidimensional phenomena: a composite indicator of social exclusion through k-means. *Soc. Indic. Res.* 159 569–591. 10.1007/s11205-021-02763-y

[B60] Lin Y. P. Fan Z. P. (2022). The relationship between rejection sensitivity and social anxiety among Chinese college students: the mediating roles of loneliness and self-esteem. *Curr. Psychol.* [Epub ahead of print]. 10.1007/s12144-021-02443-7

[B61] Liu Y. Wang M. Chang C.-H. Shi J. Zhou L. Shao R. (2015). Work-family conflict, emotional exhaustion, and displaced aggression toward others: the moderating roles of workplace interpersonal conflict and perceived managerial family support. *J. Appl. Psychol.* 100 793–808. 10.1037/a0038387 25528246

[B62] MacDonald G. Leary M. R. (2005). Why does social exclusion hurt? The relationship between social and physical pain. *Psychol. Bull.* 131 202–223. 10.1037/0033-2909.131.2.202 15740417

[B63] Magnusson K. (2018). *powerlmm: Power Analysis for Longitudinal Multilevel Models R Package Version 0.4.0.*

[B64] Malamut S. T. Garandeau C. F. Badaly D. Duong M. Schwartz D. (2022). Is aggression associated with biased perceptions of one’s acceptance and rejection in adolescence? *Dev. Psychol.* [Epub ahead of print]. 10.1037/dev0001333 35298193 PMC9274109

[B65] Mawritz M. B. Greenbaum R. L. Butts M. M. Graham K. A. (2017). I just can’t control myself: a self-regulation perspective on the abuse of deviant employees. *Acad. Manage. J.* 60 1482–1503. 10.5465/amj.2014.0409

[B66] Miyagawa Y. Niiya Y. Taniguchi J. (2021). Compassionate goals and responses to social rejection: a mediating role of self-compassion. *Curr. Psychol.* [Epub ahead of print]. 10.1007/s12144-021-02345-8

[B67] Montal-Rosenberg R. Moran S. (2022). Envy and help giving. *J. Pers. Soc. Psychol.* 122 222–243. 10.1037/pspi0000340 32852972

[B68] Norman J. B. Franco M. G. Chen J. M. (2021). Multiracial individuals’ experiences of rejection and acceptance from different racial groups and implications for life satisfaction. *J. Soc. Psychol.* [Epub ahead of print]. 10.1080/00224545.2021.1996322 34843426

[B69] Pan J. Z. Zheng X. T. Xu H. Y. Li J. Lam C. K. (2021). What if my coworker builds a better LMX? The roles of envy and coworker pride for the relationships of LMX social comparison with learning and undermining. *J. Organ. Behav.* 42 1144–1167. 10.1002/job.2549

[B70] Parks-Stamm E. J. Heilman M. E. Hearns K. A. (2008). Motivated to penalize: women’s strategic rejection of successful women. *Pers. Soc. Psychol. B* 34 237–247. 10.1177/0146167207310027 18212332

[B71] Podsakoff P. M. Mackenzie S. B. Podsakoff N. P. (2012). Sources of method bias in social science research and recommendations on how to control it. *Annu. Rev. Psychol.* 63, 539–569. 10.1146/annurev-psych-120710-100452 21838546

[B72] Podsakoff N. P. Spoelma T. M. Chawla N. Gabriel A. S. (2019). What predicts within-person variance in applied psychology constructs? An empirical examination. *J. Appl. Psychol.* 104 727–754. 10.1037/apl0000374 30640492

[B73] Poulsen J. R. Kashy D. A. (2012). Two sides of the ostracism coin: how sources and targets of social exclusion perceive themselves and one another. *Group Process. Intergr.* 15 457–470. 10.1177/1368430211430517

[B74] Preacher K. J. Selig J. P. (2012). Advantages of Monte Carlo confidence intervals for indirect effects. *Commun. Methods Meas.* 6 77–98. 10.1185/030079908x297277 18416886

[B75] Rajchert J. Konopka K. Oreziak H. Dziechciarska W. (2022). Direct and displaced aggression after exclusion: role of gender differences. *J. Soc. Psychol.* [Epub ahead of print]. 10.1080/00224545.2022.2042173 35234098

[B76] Redmond G. Main G. O’donnell A. W. Skattebol J. Woodman R. Mooney A. (2022). Who excludes? Young people’s experience of social exclusion. *J. Soc. Policy* [Epub ahead of print]. 10.1017/S0047279422000046

[B77] Reh S. Troster C. Van Quaquebeke N. (2018). Keeping (future) rivals down: temporal social comparison predicts coworker social undermining via future status threat and envy. *J. Appl. Psychol.* 103 399–415. 10.1037/apl0000281 29239645

[B78] Robinson S. L. O’Reilly J. Wang W. (2013). Invisible at work: an integrated model of workplace ostracism. *J. Manage.* 39 203–231. 10.1177/0149206312466141

[B79] Rudert S. C. Hales A. H. Buttner C. M. (2021). Stay out of our office (vs. our pub): target personality and situational context affect ostracism intentions. *J. Exp. Soc. Psychol.* 95:104142. 10.1016/j.jesp.2021.104142

[B80] Ryan R. M. Deci E. L. (2000). Self-determination theory and the facilitation of intrinsic motivation, social development, and wellbeing. *Am. Psychol.* 55 68–78. 10.1037//0003066X.55.1.68 11392867

[B81] Saco M. A. C. (2022). Inequality and exclusion in latin america: health care commodification, gendered norms, and violence. *Soc. Incl.* 10 1–4. 10.17645/si.v10i1.5240

[B82] Santor D. A. Yazbek A. A. (2006). Soliciting unfavourable social comparison: effects of self-criticism. *Pers. Indiv. Differ.* 40 545–556. 10.1016/j.paid.2005.06.029

[B83] Schock A.-K. Gruber F. M. Scherndl T. Ortner T. M. (2019). Tempering agency with communion increases women’s leadership emergence in all-women groups: evidence for role congruity theory in a field setting. *Leadersh. Q.* 30 189–198. 10.1016/j.leaqua.2018.08.003

[B84] Scott W. Tyser J. Penningroth S. L. Strauch C. (2022). Assessing self-schema content: the relationship of psychological needs to early maladaptive schemas, rejection sensitivity, and personality traits. *Self Identity* 21 317–338. 10.1080/15298868.2021.1895882

[B85] Stinson D. A. Logel C. Shepherd S. Zanna M. P. (2011). Rewriting the self-fulfilling prophecy of social rejection: self-affirmation improves relational security and social behavior up to 2 months later. *Psychol. Sci.* 22 1145–1149. 10.1177/0956797611417725 21813799

[B86] Suls J. Martin R. Wheeler L. (2000). Three kinds of opinion comparison: the triadic model. *Pers. Soc. Psychol. Rev.* 4 219–237. 10.1207/S15327957pspr0403_2

[B87] Sun J. Li Y. Li S. Li W.-D. Liden R. C. Zhang X. (2021). Unintended consequences of being proactive? Linking proactive personality to coworker envy, helping, and undermining, and the moderating role of prosocial motivation. *J. Appl. Psychol.* 106 250–267. 10.1037/apl0000494 32297764

[B88] Tai K. Narayanan J. McAllister D. J. (2012). Envy as pain: rethinking the nature of envy and its implications for employees and organizations. *Acad. Manage. Rev.* 37 107–129. 10.5465/amr.2009.0484

[B89] Tang P. M. Yam K. C. Koopman J. Ilies R. (2022). Admired and disgusted? Third parties’ paradoxical emotional reactions and behavioral consequences towards others’ unethical pro-organizational behavior. *Pers. Psychol.* 75 33–67. 10.1111/peps.12446

[B90] Taylor S. E. Lobel M. (1989). Social-comparison activity under threat - downward evaluation and upward contacts. *Psychol. Rev.* 96 569–575. 10.1037/0033-295x.96.4.569 2678204

[B91] Tussing D. V. Wihler A. Astandu T. V. Menges J. I. (2022). Should I stay or should I go? The role of individual strivings in shaping the relationship between envy and avoidance behaviors at work. *J. Organ. Behav.* 43 567–583. 10.1002/job.2593

[B92] Vecchio R. P. (2005). Explorations in employee envy: feeling envious and feeling envied. *Cogn. Emot.* 19 69–81. 10.1080/02699930441000148

[B93] Vial A. C. Brescoll V. L. Napier J. L. Dovidio J. F. Tyler T. R. (2018). Differential support for female supervisors among men and women. *J. Appl. Psychol.* 103 215–227. 10.1037/apl0000258 28933911

[B94] Wang Q. Tu R. L. Hu W. Luo X. Zhao F. Q. (2021). Childhood psychological maltreatment and depression among chinese adolescents: multiple mediating roles of perceived ostracism and core self-evaluation. *Int. J. Environ. Res. Public Health* 18:11283. 10.3390/ijerph182111283 34769803 PMC8583377

[B95] Wang Z. Jetten J. Steffens N. K. (2022). Restless in an unequal world: economic inequality fuels the desire for wealth and status. *Pers. Soc. Psychol. Bull.* [Epub ahead of print]. 10.1177/01461672221083747 35373639

[B96] Watson D. Clark L. A. Tellegen A. (1988). Development and validation of brief measures of positive and negative affect: the PANAS scales. *J. Pers. Soc. Psychol.* 54 1063–1070. 10.1037/0022-3514.54.6.1063 3397865

[B97] Yaakobi E. (2021). Personality as a moderator of immediate and delayed ostracism distress. *Br. J. Soc. Psychol.* [Epub ahead of print]. 10.1111/bjso.12484 34287977

[B98] Yang C. Tang R. X. (2021). Validating the “two faces” of envy: the effect of self-control. *Front. Psychol.* 12:731451. 10.3389/fpsyg.2021.731451 34777112 PMC8578062

[B99] Yu L. T. Duffy M. K. Tepper B. J. (2018). Consequences of downward envy: a model of self-esteem threat, abusive supervision, and supervisory leader self-improvement. *Acad. Manage. J.* 61 2296–2318. 10.5465/amj.2015.0183

[B100] Zhang Y. J. Bolino M. C. Yin K. (2022). The interactive effect of perceived overqualification and peer overqualification on peer ostracism and work meaningfulness. *J. Bus. Ethics* [Epub ahead of print]. 10.1007/s10551-021-05018-5

